# Double Relapsed and/or Refractory Multiple Myeloma: Clinical Outcomes and Real World Healthcare Costs

**DOI:** 10.1371/journal.pone.0136207

**Published:** 2015-09-14

**Authors:** Sarah Gooding, I-Jun Lau, Mimi Sheikh, Pamela Roberts, Julia Wong, Emmy Dickens, Ash Bullement, Jamie Elvidge, Dawn Lee, Karthik Ramasamy

**Affiliations:** 1 Department of Haematology, Oxford University Hospitals NHS Trust, Churchill Hospital, Old Road, Headington, Oxford, OX3 7LE, United Kingdom; 2 National Institute for Health Research (NIHR) Oxford Biomedical Research Centre, Oxford University Hospitals NHS Trust, Oxford, OX3 7LE, United Kingdom; 3 MRC Human Immunology Unit, University of Oxford, Weatherall Institute of Molecular Medicine, Headley Way, Oxford, OX3 9DS, United Kingdom; 4 Department of Oncology, University of Oxford, Old Road Campus Research Building, Oxford University Old Road Campus, Oxford, OX3 7LH, United Kingdom; 5 BresMed Health Solutions, 84 Queen Street, North Church House, Sheffield, S1 2DW, United Kingdom; 6 Royal Berkshire Hospital NHS Trust, London Rd, Reading, RG1 5AN, United Kingdom; ACTREC (Advanced Centre for Treatment, Research and Education in Cancer) / Tata Memorial Centre, INDIA

## Abstract

Double relapsed and/or refractory multiple myeloma (DRMM), MM that is relapsed and/or refractory to bortezomib and lenalidomide, carries a poor prognosis. The healthcare costs of DRMM have not previously been reported. We analyzed detailed medical resource utilization (MRU) costs, drug costs and outcomes for 39 UK patients receiving standard DRMM therapy. Median OS in this cohort was 5.6 months. The mean cost of DRMM treatment plus MRU until death was £23,472 [range: £1,411–£90,262], split between drug costs £11,191 and other resource use costs £12,281. The cost per assumed quality-adjusted life year (QALY) during DRMM was £66,983. These data provide a standard of care comparison when evaluating the cost-effectiveness of new drugs in DRMM.

## Introduction

Patients with treatment-refractory malignancy have poor outcomes and high healthcare costs. In Multiple Myeloma (MM), the introduction of the proteasome inhibitor bortezomib and immunomodulatory drugs (IMiDs) thalidomide and lenalidomide has improved survival over the last decade [1], but increased the cost of treatment. While these drugs can result in remission, most patients will relapse with increasing symptom burden and worsening prognosis [2]. Double relapsed and/or refractory multiple myeloma (DRMM), MM that is relapsed and/or refractory to bortezomib and lenalidomide [3], carries a poor prognosis and therapeutic options remain limited. An IMWG retrospective analysis of patients who relapsed following bortezomib and at least one of the IMiDs showed a median overall survival (OS) and progression-free survival (PFS) of 9 months and 5 months respectively [4]. Only those potentially eligible for further clinical trials with Eastern Cooperative Oncology Group Performance Status (ECOG PS) 0–2 were included, indicating survival may have been overestimated relative to the entirety of this heavily treated population.

Studies of healthcare costs for relapsed and/or refractory MM patients receiving bortezomib and/or lenalidomide based regimens have been undertaken in a variety of countries, using ‘real-world’ data and/or economic modelling, often from patients enrolled in clinical trials [5–9]. However, none of them address MRU for patients relapsed after bortezomib and lenalidomide. The third-generation IMiD pomalidomide and second-generation proteasome inhibitor carfilzomib have recently been licensed and have efficacy in DRMM [10]. The cost burden and clinical outcomes outside of trials of this phase of the disease has never been established, so new therapies with efficacy in DRMM have no published benchmark against which to judge cost-effectiveness. To ensure resources are allocated appropriately, the cost-effectiveness evaluation of these therapies in the DRMM setting must involve non-clinical trial, real world MRU data from relevant patients. Our data provides a standard of care comparison when evaluating cost-effectiveness of new drugs in DRMM.

## Methods

### Ethics Statement

All patients whose data were included in this study had provided written consent for the use of their anonymised data for the purposes of audit and service improvement by the Thames Valley Cancer Network, UK. This study was appropriately registered using the clinical audit project proposal system at Oxford University Hospitals NHS Trust, UK. The Clinical Audit Lead reviewed and approved the proposal, and in line with the UK NHS National Research Ethics Service (NRES) guidance, deemed it not to require IRB or ethics committee approval.

Anonymised data on clinical outcomes, anti-myeloma therapies prescribed and MRU were obtained for 39 DRMM patients pre-treated with or intolerant to bortezomib and lenalidomide in the Thames Valley Cancer Network, UK from 2011 to 2014 (Table A in S1 File). Based on the UK National Institute for Health and Care Excellence (NICE) guidelines, bortezomib-based therapy was used for first relapse unless contraindicated. Lenalidomide-dexamethasone combination was approved for second and subsequent relapse. Relapsed myeloma patients were identified using pharmacy-generated lists of all sequential lenalidomide recipients between January 2011 and July 2013 at Oxford University Hospitals and the Royal Berkshire Hospital, Reading, UK. The strategy of using hospital pharmacy dispensing data to identify subjects ensured all lenalidomide recipients during the study period were included in the analysis, as long as they had Multiple Myeloma and had progressed on or were refractory to lenalidomide, according to IMWG criteria [11]. 34 (87%) patients were pre-treated with lenalidomide and bortezomib, and had relapsed following, or failed to tolerate both therapies. 5 patients had not received bortezomib due to pre-existing peripheral neuropathy, sufficiently severe to contraindicate its use.

OS and PFS were calculated from the start date of the first therapy following relapse after lenalidomide (‘1^st^ DRMM therapy’) until either IMWG criteria for progression/relapse were reached or death. The data censor date was 10 January 2014 for patients still alive. For each patient the following occurrences of MRU were retrieved from health care records from the start of each successive DRMM therapy until death or censoring: detailed drug regimens, outpatient clinic and chemotherapy unit attendances, inpatient/hospice admissions, supportive therapies, medical procedures, radiological investigations, blood product transfusions and blood tests. Costings were calculated using NHS reference costs 2012–13. These were combined to give one MRU cost from the start of DRMM therapy to death or censoring, using a micro-costing approach. Drug costs were separately calculated for each successive DRMM therapy.

## Results

When offered DRMM therapy, 67% of patients preferred active treatment to palliative care (Table 1). The median age of those who chose palliative care was 73.2 years compared to 59.9 for active therapy (p<0.001). The first DRMM treatment regimen was typically bendamustine, thalidomide and dexamethasone (43.6%) as published previously [12]. Retreatment with bortezomib (15.4%) or lenalidomide (33.3%) based regimens was used if poor bone marrow reserve precluded bendamustine use and suitable clinical trial alternatives were lacking.

**Table 1:** Patient characteristics.

**Median age at diagnosis, years (range)** *	64.3 (45–79)
**Median age by treatment intent: Active/ Palliative**	59.9 (n = 26)/ 73.2 (n = 13)
**Isotope: IgA/ IgG/ Light chain**	7 (51.3%)/ 20 (18.0%)/ 12 (30.8%)
**ISS stage I at diagnosis**	6 (15.4%)
**ISS stage II at diagnosis**	9 (23.1%)
**ISS stage III at diagnosis**	10 (25.6%)
**ISS stage at diagnosis unknown**	14 (35.9%)
**Previous thalidomide-based treatment**	34 (87.2%)
**Previous bortezomib-based treatment**	34 (87.2%)
**Previous lenalidomide-based treatment**	39 (100.0%)
**Previous high dose melphalan with stem cell rescue**	17 (43.5%)
**Previous additional alternative treatment(s):**	
Vincristine-based regime	7 (17.9%)
Melphalan-based regime	5 (12.8%)
Allograft	1 (2.6%)
Median years from diagnosis to DRMM point (range)*	4 years 9.5 months (6.5 mo– 10.5 y)
**Median number regimes prior to DRMM (range)**	4 (2–5)
**1st DRMM therapy contained bendamustine**	17 (43.6%)
**1st DRMM therapy contained bortezomib**	6 (15.4%)
**1st DRMM therapy DT-PACE**	1 (2.6%)
**1st DRMM therapy contained lenalidomide**	13 (33.3%)
**No treatment given at DRMM**	2 (5.1%)
**2** ^**nd**^ **DRMM therapy (n = 7):**	
Bendamustine, thalidomide, dexamethasone	2 (28.6%)
Melphalan, dexamethasone	2 (28.6%)
Thalidomide-based regime	2 (28.6%)
Pomalidomide, dexamethasone	1 (14.3%)
**3** ^**rd**^ **DRMM therapy (n = 4):**	
Bortezomib, cyclophosphamide, dexamethasone	2 (50.0%)
Bortezomib, melphalan, prednisone	1 (25.0%)
Bendamustine, thalidomide, dexamethasone	1 (25.0%)
**Response to 1** ^**st**^ **DRMM therapy:**	
CR	1 (2.6%)
VGPR	2 (5.1%)
PR	8 (20.5%)
SD	13 (33.3%)
PD or death within first month	14 (35.9%)
Unknown	1 (2.6%)
**Duration of treatment (SD)**	104.9 days (63.8)
**Response to 2** ^**nd**^ **DRMM therapy:**	
PR	3 (42.9%)
SD	2 (28.6%)
PD or death within first month	2 (28.6%)
**Response to 3** ^**rd**^ **DRMM therapy:**	
PR	2 (50.0%)
PD or death within first month	1 (25.0%)
Unknown	1 (25.0%)

*Date of diagnosis unavailable in 2 cases. ISS: International Staging System; DRMM: Double Relapsed and/or Refractory Multiple Myeloma; CR: Complete response; VGPR: Very good partial response; PR: Partial response; SD: Stable disease; PD: Progressive disease [11]

Regimen choice was made by the treating clinician and based on ECOG PS and response/ side effect profile of prior therapies. Third-generation IMiDs and second-generation proteasome inhibitors were not routinely available to this cohort, excepting pomalidomide in one patient. Two patients who had not previously received bortezomib due to neuropathy did receive it at DRMM with no recorded worsening of neuropathy; one had progressive disease despite its use and the other had a partial response of 6 months duration.

**Fig 1:**
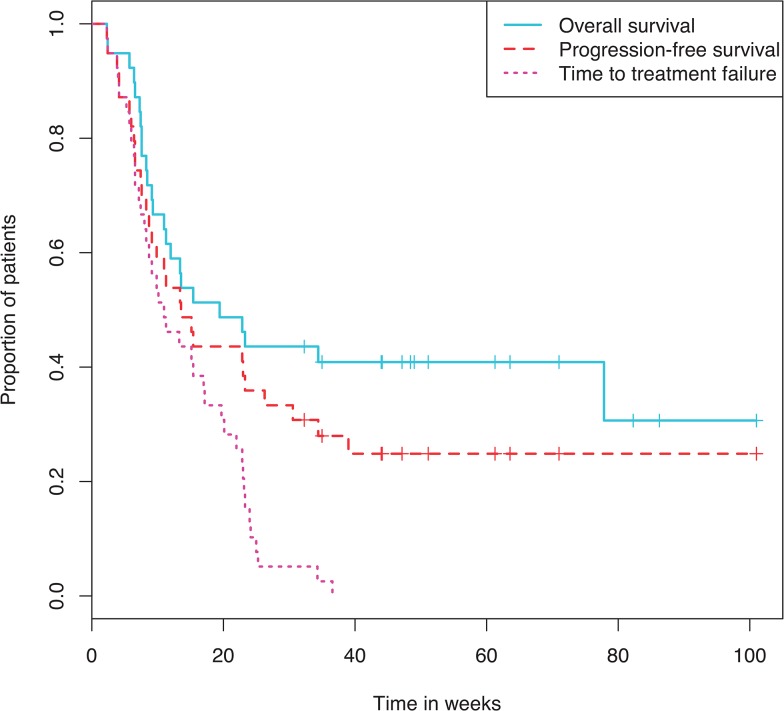
Kaplan Meier curve showing overall survival, progression free survival and time to treatment failure.

Median PFS was 5.2 months and median OS was 5.6 months from start of DRMM therapy (Fig 1). These statistics reflect a steep drop in survival early on with a few patients surviving significantly longer. 24/39 (61.5%) patients had died by the end of follow-up, of whom 79% died in hospital/ hospice. The cohort was deemed too small and heterogeneous for analysis of regimen effect on survival.

MRU was high in this cohort in comparison with that usually observed at earlier lines of therapy (Table 2). 60 inpatient hospital admissions occurred during DRMM therapy in 39 patients. Admissions lasted 9.3 days on average, for indications including pain management, renal failure and most commonly neutropenic fever, with some cases of culture-confirmed septicaemia. Prolonged inpatient admissions, frequent AEs and high transfusion requirements reflect a poor quality of life (QoL) among these patients (Table 3). The most common recorded Grade 3–4 adverse events (AEs) were anaemia (43.6%), thrombocytopenia (28.2%) and bone pain (33.3%) (Table 3, Table B in S1 File).

**Table 2:** Medical Resource Utilisation Costs of Double Relapsed and/or Refractory Multiple Myeloma Therapy.

**MRU Category**	**Treatment given**	**Occurrences**		**Cost per Patient**	
***Drug costs***					
	Bortezomib-based	6/39		£4,022	
	Lenalidomide-based	13/39		£3,913	
**1** ^**st**^ **DRMM therapy** (per 28 day cycle)^a^	DT-PACE	1/39		£946	
	Bendamustine-based	17/39		£1,332	
	No active treatment	2/39		£0	
	Average			£2,532	
	Ben/ Thal/ Dex	2/39		£853	
	Mel/ Dex	2/39		£133	
**2** ^**nd**^ **DRMM therapy** (per 28 day cycle) ^a^	Thal	2/39		£298	
	Pom/ Dex	1/39		£8,887	
	No 2^nd^ DRMM therapy	32/39		£0	
	Average			£294	
	Bor/ Cyc/ Dex	2/39		£4,118	
**3** ^**rd**^ **DRMM therapy** (per 28 day cycle) ^a^	Bor/ Mel/ Pred	1/39		£3,847	
	Ben/ Thal/ Dex	1/39		£1,983	
	No 3^rd^ DRMM therapy	35/39		£0	
	Average			£361	
Total 1^st^ DRMM therapy drug costs (all cycles)				£9,527	
Total 2^nd^ DRMM therapy drug costs (all cycles)				£807	
Total 3^rd^ DRMM therapy drug costs (all cycles)				£857	
**Total drug costs (all cycles)**				**£11,191**	
		**Occurrences during DRMM period** ^**d**^			
**MRU Category**	**MRU Item**			**Cost per Patient**	**Range**
		**Mean**	**SD**		
***Other MRU costs***					
**Inpatient admissions**	Night as inpatient	9.3	7.9	£2,463	£0, £16,169
	Outpatient	4.2	4.1	£630	£0, £3,012
**Attendances**	Day therapy unit	12.8	9.7	£4,331	£0, £11,848
	Triage, not admitted	0.4	0.9	£46	£0, £344
	CT scan	0.4	0.8	£42	£0, £435
**Invasive and radiological procedures**	MRI scan	0.3	0.6	£57	£0, £343
	X-ray	1.1	2.7	£31	£0, £311
	Maxillofacial	0.1	0.2	£21	£0, £406
	Other^b^	0.3	0.5	£172	£0, £3,225
**Supportive therapy**	Bisphosphonate^c^	2.6	2.5	£217	£0, £713
	Radiotherapy	1.1	3.6	£1,237	£0, £21,643
**Transfusion**	Red blood cells (units)	5.9	6.0	£1,684	£0, £5,993
	Platelets (units)	2.3	3.8	£1,200	£0, £6,784
	Full blood count	21.6	13.0	£65	£0, £196
**Blood tests**	Biochemistry	20.6	15.6	£26	£0, £110
	Immunology	4.4	2.9	£22	£0, £60
	Microbiology	5.6	7.0	£38	£0, £278
**Total other MRU costs**				**£12,281**	**£995,**
					**£40,274**
**Total (drug costs and other MRU costs)**				**£23,472**	**£1,411,**
					**£90,262**

MRU: Medical Resource Utilization; DRMM: Double Relapsed and/or Refractory Multiple Myeloma; Ben: Bendamustine; Thal: Thalidomide; Dex: Dexamethasone; Mel: Melphalan; Pom: Pomalidomide; Bor: Bortezomib; Cyc: Cyclophosphamide; Pred: Prednisone.

^a^Drug costs have been calculated as the average of all patients undertaking each regimen. Dosing changes have been incorporated where provided. Additional dosing regimen details have been taken from product SPCs. Costs are taken from BNF or eMIT; and are applied using the appropriate pack/ vial size. The average surface area of a patient (used for IV therapies) is taken from the MM-003 clinical trial (approximately 1.86m^2^). Differences in costs of the same treatment between treatment lines are caused by differences in dosing for individual patients.

^b^Other medical procedures consisted of: 1 vertebroplasty; 1 facet joint injection; 1 endoscopy; 1 bronchoscopy; 1 hip fracture repair under general anaesthetic; 2 PET scans and 4 ultrasound scans.

^c^Bisphosphonate costs calculated assuming all patients on bisphosphonates are on an average dose. The figure shows the approximate number of cycles for which patients are on bisphosphonate treatment.

^d^All MRU occurrences were recorded from initiation of 1^st^ DRMM therapy until the end of follow up (or death)

**Table 3:** Surrogates of Quality of Life during Double Relapsed and/or Refractory Multiple Myeloma Therapy.

Surrogates of Quality of Life^a^	Number of patients
**Grade 3–4 Adverse events during DRMM therapy:** ^**b**^	
Anaemia	17 (43.6%)
Neutropaenia	7 (17.9%)
Thrombocytopaenia	11 (28.2%)
Bleeding	1 (2.6%)
Febrile neutropaenia	6 (15.4%)
Bone pain	13 (33.3%)
Acute renal failure	4 (10.3%)
Dehydration / vomiting / diarrhoea	2 (5.1%)
**Admissions during DRMM therapy (SD)**	1.3 per patient (1.1)
**Duration of admissions (SD)**	9.3 days (7.9)
**Outpatient clinic appointments during DRMM therapy (SD)**	4.2 per patient (4.1)
**Day therapy unit visits during DRMM, including CT & MRI (SD)**	12.2 per patient (9.7)
**RBC units during DRMM therapy (SD)**	5.9 per patient (6.0)
**Platelet units during DRMM therapy (SD)**	2.3 per patient (3.8)

^a^All Surrogates of Quality of Life were recorded from initiation of 1^st^ DRMM therapy until the end of follow up (or death)

^b^Adverse events with no recorded grade are assumed to be grade 3 or 4.

The mean total MRU cost per patient from start of DRMM therapy until death or censor was £12,281 (Table 2). This comprises: clinic attendances £5,007 (41%); inpatient admissions £2,884 (20%); transfusions £2,479 (23%); supportive therapy £1,454 (12%); radiology/procedures £323 (3%); blood tests £151 (1%). The mean drug cost of DRMM therapy is estimated to be £11,191 per patient. The mean total cost of treatment plus MRU is therefore £23,472 [range: £1,411 - £90,262]; £760 per week of life with DRMM. Formal QoL data is lacking in this retrospective cohort but has been previously published for DRMM patients in the MM-003 trial [13]. Assuming that QoL was the same in this cohort (utility 0.59) and remained constant throughout patients’ lifetimes, our analysis indicated a cost per QALY of £66,983.

## Conclusion

Although the small sample size of this cohort limits the ability to draw definitive survival conclusions, PFS and OS were poor, with a wide range due to the inclusion of all patients whether treated with active or palliative intent, but were similar to published examples [4]. Despite the poor outcomes, up to two thirds of DRMM patients want to be treated with active intent to improve survival, reiterating the need to develop therapies that give patients an improved prognosis, whilst being cost-effective and well tolerated in a heavily pre-treated patient group. The heterogeneity of this cohort is acknowledged but intentional, representing a typical real world hospital cohort of patients, where a range of therapeutic options must be employed, constrained by varying patient-related factors such as ECOG PS, drug tolerance, social situation and patient choice.

This is the first report of non-clinical trial based ‘real-world’ MRU cost analysis in the setting of DRMM. These patients have high MRU costs. A comparable cost analysis report relates to a subset of 54 ‘4^th^ line’ real world relapsed/refractory patients in a Netherlands study, recruited from a previous trial cohort [7]. However, it is likely that this cohort (data collected from 2001 to 2009) included patients who received bortezomib (n = 12) and/or lenalidomide (n = 20) for the first time at 4^th^ line, as they were first made available during the study period. At €32,889 per patient (range: €1,055–€144,967), costs reported in that study are comparable to our findings. However an estimated cost per QALY of £66,983 is a significant increase on that reported by Brown et al in a UK study of the cost effectiveness of Lenalidomide-based therapies after one prior therapy [5], where cost was £30,153/QALY. The difference reflects the limited benefit to survival of any current therapy at DRMM, and the higher MRU costs at this later stage of disease.

Compared with a decade ago, the price range of new anticancer agents has more than doubled [14], and the cost of care analysis of these agents is imperative. New MM therapies carfilzomib and pomalidomide have been priced significantly higher than currently available anti-myeloma drugs. Any subsequent cost benefit analysis comparisons performed in DRMM patients must be set in the context of the high background MRU as observed in our cohort. It is highly relevant that the cost per assumed QALY in this cohort is double that usually accepted by the UK National Institute for Health and Care Excellence [15]. In addition to improving survival, therapies that induce higher response rates or arrest disease progression could potentially increase therapy costs, but lower MRU costs and improve QoL if progression is halted. Biomarkers that focus use of new drugs to cohorts of patients where maximum benefit is obtained would improve cost-effectiveness further. New treatments should be compared with real-world non-trial outcomes such as that provided here, to give a realistic picture of the value of these new drugs.

## Supporting Information

S1 File Table A: Complete Data collected (excepting adverse events) on 39 patients with Double Refractory Multiple Myeloma. Table B: Adverse Events recorded for 39 patients with Double Refractory Multiple Myeloma. (XLS)
